# Supplementary material for: A Critical View of the Application of the APEX Software (Aqueous Photochemistry of Environmentally-Occurring Xenobiotics) to Predict Photoreaction Kinetics in Surface Freshwaters
Source: Molecules. 2019 Dec 18;25(1):9. doi: 10.3390/molecules25010009 (PMC7017383; doi:10.3390/molecules25010009)
Supplement: Supplementary file 1 [file molecules-25-00009-s001.zip › 3.2.4_gcc-4.4.0.zip!/3.2.4_gcc-4.4.0/3.2.4_gcc-4.4.0/doc/octave/html/interpreter/Bar-series.html]

Bar series - Untitled


Next: Contour groups,
Previous: Area series,
Up: Object Groups


---

##### 15.2.8.3 Bar series

Bar series objects are created by the `bar` or `barh`
functions. Each `hggroup` element contains a single patch object.
The properties of the bar series are

`showbaseline` `baseline` `basevalue`: The property `showbaseline` flags whether the baseline of the bar series is displayed (default is "on"). The handle of the graphics object representing the baseline is given by the `baseline` property and the y-value of the baseline by the `basevalue` property. Changes to any of these property are propagated to the other members of the bar series and to the baseline itself. Equally changes in the properties of the base line itself are propagated to the members of the corresponding bar series. `barwidth` `barlayout` `horizontal`: The property `barwidth` is the width of the bar corresponding to the width variable passed to `bar` or barh. Whether the bar series is "grouped" or "stacked" is determined by the `barlayout` property and whether the bars are horizontal or vertical by the `horizontal` property. Changes to any of these property are propagated to the other members of the bar series. `linewidth` `linestyle`: The line width and style of the edge of the patch objects making up the bars. See Line Styles. `edgecolor` `facecolor`: The line and fill color of the patch objects making up the bars. See Colors. `xdata`: The nominal x positions of the bars. Changes in this property and propagated to the other members of the bar series. `ydata`: The y value of the bars in the `hggroup`. `xdatasource` `ydatasource`: Data source variables.
